# Surgical outcomes in patients with epilepsy after viral encephalitis: contribution of SEEG study

**DOI:** 10.1186/s12883-019-1396-1

**Published:** 2019-07-17

**Authors:** Yi-Ou Liu, Wen-Jing Zhou, Bo Hong, Tong Zhao, Yue-feng Wang

**Affiliations:** 1 Department of Epilepsy Center, Division of Neurology, Tsinghua University Yuquan Hospital, Beijing, China; 2 Department of Biomedical Engineering, School of Medicine, Tsinghua University, Beijing, China; 3 Department of Pathology, Tsinghua University Yuquan Hospital, Beijing, China

**Keywords:** Surgical outcome, Epilepsy, Viral encephalitis, Stereoelectroencephalography (SEEG), Epileptogenicity index (EI)

## Abstract

**Background:**

Nowadays, few surgery analysis has been reported in cases of epilepsy after viral encephalitis(VE). Herein, this study was to evaluate the efficacy of surgery and capability of stereoelectroencephalography (SEEG) in the definition of the epileptogenic zone (EZ) after VE, and also to explore the relationship between the SEEG features and the surgical outcomes.

**Methods:**

We retrospectively analyzed 10 surgically treated patients that identified to suffer from epilepsy secondary to VE using SEEG, and investigated the SEEG features associated with surgical outcomes in these patients. Besides visual analysis, we used the epileptogenicity index (EI), a semi-quantitative and supplementary tool to evaluate the validity of SEEG in the context of VE.

**Results:**

Among the 10 operated patients, 3 of them became completely seizure-free. The patients who got totally seizure free or significant improvement, the seizure onset was located either in the antero-mesial temporal structures or focal gyrus; patients who got worthwhile improvement or no improvement, the seizure started from multiple brain lobes. The number of electrodes classified as epileptogenic visually involved were closely correlated with EI positive onses.Anatomic areas defined and shown as EZ on MRI by visual assessment were also defined as epileptogenic by the EI in these cases.

**Conclusion:**

Apart from exploring the surgical outcome related to epilepsy after VE, we also bring insight into the relationship between the SEEG features and surgical outcome with the application of the supplementary methods.

## Background

Viral encephalitis (VE) is a process of acute inflammation of the brain parenchyma caused by viral infection, which is the most frequent cause of encephalitis in Asia [1, 2]. The epilepsy associated with VE is often drug resistant, and the epileptic seizures may occur during and after the acute phase of VE. Despite the high rates of pharmacoresistance in epilepsy after viral infection, there is little coverage of this issue, and this aetiology is still under-represented in epilepsy [3]. The surgical analysis in cases of epilepsy post encephalitis is rare due to the difficulties in precise location of epilepsy. Most pharmacoresistant epilepsy, especially after VE, are associated with a diffuse MRI/CT-identifiable structural lesion after cerebral infection, which is hard to be resected [4]. These patients may be excluded from surgery. There are controversial views in previous literature on this issue: in certain subgroups of patients with epilepsy, the surgical outcomes after VE were encouraging, whereas some surgical outcomes were generally unsatisfactory [5]. Previous studies have demonstrated that patients with epilepsy following meningitis and encephalitis may have a favorable surgical outcome if they are had mesial temporal sclerosis (MTS) [6, 7]. In these patients, surgery is considered as the treatment of choice, but it is a great challenge for defining the EZ. In the presurgical evaluation of these patients, scalp electroencephalogram (EEG) studies are often inadequate and requires invasive techniques. SEEG allows sampling from deep cortical tissues and regions beyond the reach of subdural electrodes, which also provide direct intralesional recordings [8–10]. Few human data are available with the application of the intracerebral EEG recordings within suspected epileptogenic zone after VE, and there were rare studies on the characteristics of SEEG after VE and its correlation with surgical efficacy. In this present study, we reviewed SEEG data in a group of patients with epilepsy after VE, and the objective of which was to evaluate the applicability of SEEG in determining the EZ after VE. Additionally, our objectives were also to assess the electro-clinical phenotype and the underlying epileptogenic zone defined by SEEG recordings, meanwhile, to reveal the relationship between the SEEG features and the surgical outcomes.

In addition to visual judgement, we also used the EI, a semi-quantitative analysis designed for SEEG data, which can potentially supplement the standard visual analysis of the SEEG data by clinicians and evaluated the validity of this technique in the context of VE. Furthermore, we aimed to explore the relationship between EZ features (including visual evaluation and EI analysis) and surgical efficacy.

## Methods

### Patients and methods

A total of 150 patients underwent epilepsy surgery using SEEG between January 2014 and March 2017 at the Epilepsy Center of Yuquan Hospital Tsinghua University in Beijing, among which we retrospectively enrolled 10 patients who suffered from epilepsy secondary to VE. encephalitis defined by presentation with altered mental Status lasting more than 24 h, with at least three of the following manifestations:(1) temperature ≥ 38 °C within 72 h,(2) new-onset generalized or focal seizures, (3) new-onset focal neurologic deficits, (4) cerebrospinal fluid (CSF) white blood count (WBC) count ≥5/mm3, (5) abnormality on neuroimaging consistent with encephalitis, (6) abnormality on electroencephalography (EEG) [11]. Patients were categorized as VE only if they had a positive antibody titer in the CSF, a positive polymerase chain reaction (PCR) findings. There is currently no consensus definition for postencephalitic epilepsy (PE), with most studies applying the use of ongoing AEDs as their definition of PE, with variable time thresholds [12]. Our operational definition of PE was defined by the recurrent seizures required long-term AEDs for more than 12 months after acute episode of encephalitis. All patients had a comprehensive presurgical evaluation including MRI, long-term video-EEG (VEEG), PET-scan and neuropsychological test. The MRI scans of patients were performed using 3 Telsa (3 T) magnet. Three-dimensional (3D) volume fast-field echo T1-weighted images and FLAIR image were obtained, and other sequence required were obtained from the reconstruction. FDG-PET was superimposed on the 3D brain MRI in order to determine the potential alterations of the cerebral metabolism .

### Acquisition and analysis of SEEG data

Since the noninvasive investigations did not allow a clear definition of the EZ,SEEG recordings was necessary in these patients. The placement of electrodes was guided in each patient by the hypotheses for EZ localization. Between 8 and 18 electrodes were implanted per patient depending on the suspected origins. The number and location of electrodes implanted varied on the basis of ictal symptoms, interictal spiking and ictal onset (scalp EEG), MRI, and PET, SEEG recordings were performed using multiple contact electrodes (10–15 contacts, length, 2 mm, diameter, 0.8 mm, 1.5 mm apart) placed intracerebrally according to Talairach’s stereotactic [13]. We decided to proceed with SEEG in our patients for the following reasons: i) bilateral lesions on MRI (pt no. 1,4,9), especially for Patient 4 to protect the functional area by limiting the removal range; ii) although symptomology and PET-CT are instructive, MRI does not have a clear focus, the onset of the seizure is not clear or there is inconsistency between scalp EEG data and clinical manifestation, so it is impossible to locate accurately (pt no. 2,6,8,10); iii) The onset of the seizure is consistent with that of the interictal discharges, but MRI does not have a definite lesion (pt no.5,7); iv)there showed a definite lesion on MRI,but scalp EEG recordings did not identify position involved first during the seizures precisely, and the extent of the removal should also be defined(pt no.3). Afterwards, a postoperative computed tomography (CT) scan was obtained,and a CT/MRI data fusion was performed to check the precise location of each electrode [14]. The signals were recorded on a 256 channels NeuroFax software (MEE-1000; Nihon Kohden, Tokyo, Japan) system. The interictal epileptiform discharges (IEDs), slow waves, fast activities, as well as high freguency oscillationos(HFO) were observed. When necessary, SEEG recordings was prolonged in order to record patients’ habitual seizures. All seizures were visually reviewed by two independent reviewers. We defined the ictal onset of first changes of the EEG manifestation in each seizure, which is characterized by low voltage fast activities, amplitude decrement or repetitive spikes [15].

### Signal analysis: EI

**Fig. 1:**
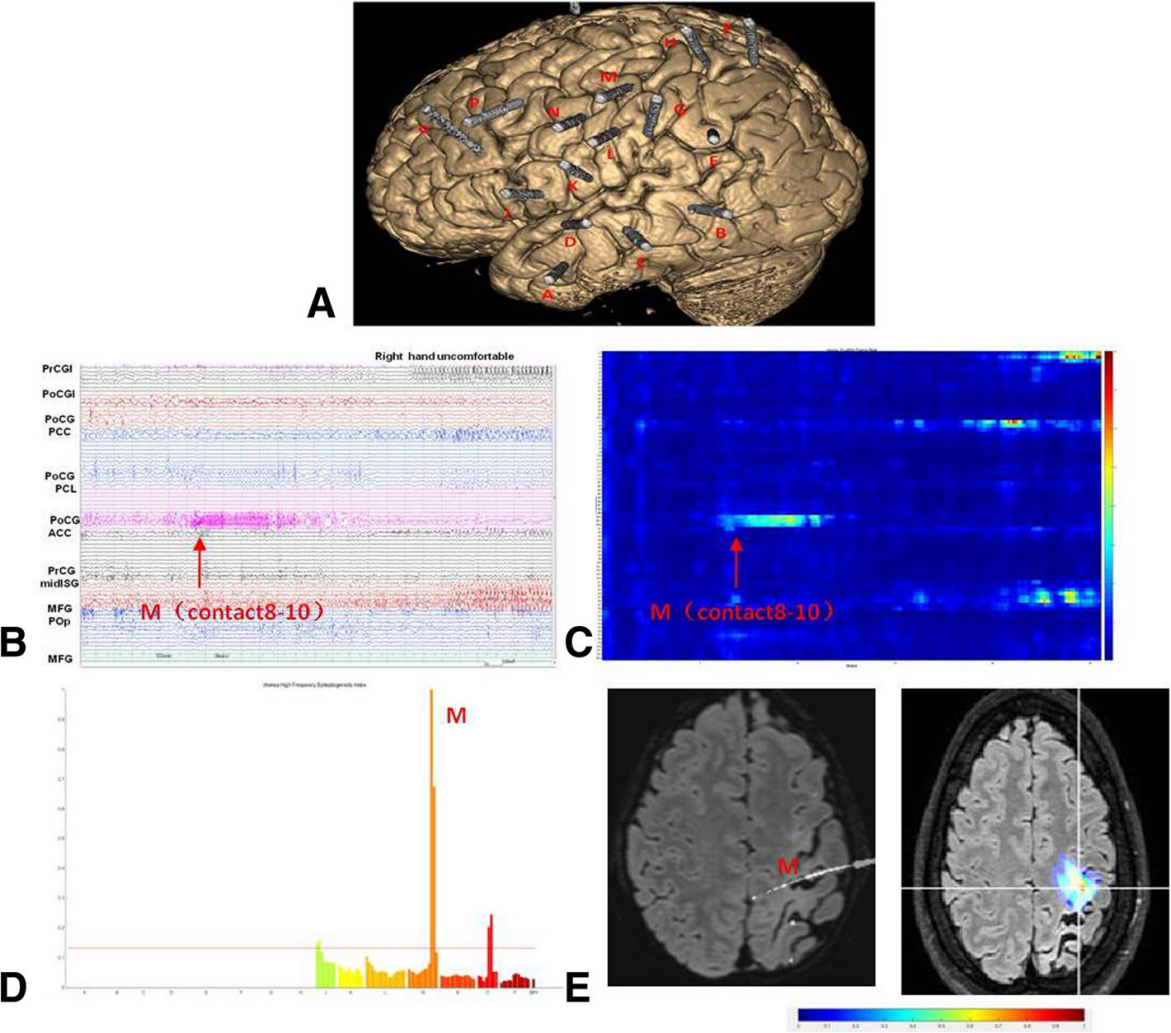
SEEG recordings of Patient 3. **a** Location of the SEEG electrodes shown on the 3D MRI image, The electrodes mainy explored perisylvian region of left hemisphere. **b** Seizures started with low voltage fast activities in the postcentral gyrus (external leads of electrode M). **c** Axis X indicates time of selected seizure, axis y indicates electrodes, ER(Energy Ratio)of high frequencies changes was depicted in a color scale (From red to blue ER intensity decreased), Quantification of the seizure onset was in the same position(electrode M) as visually seen. **d** Axis X indicates electrodesrodes, axis Y indicates EI values, highest EI quoted on the chart demonstrating maximal epileptogenicity in the electrode M. **e** EI values were superimposed on the axial planes of MRI, (color scale:From red to blue EI value decreased),seizure onset was confined to a single gyrus. PrCGI:precentral gyrus of insular; PoCGI:postcental gyrus of insular; PCC:posterior cingular cortex; PCL:paracentral lobule; PoCG:postcentral gyrus; PrCG:precentral gyrus; Pop:parietal operculum MFG:middle frontal gyrus

**Fig. 2:**
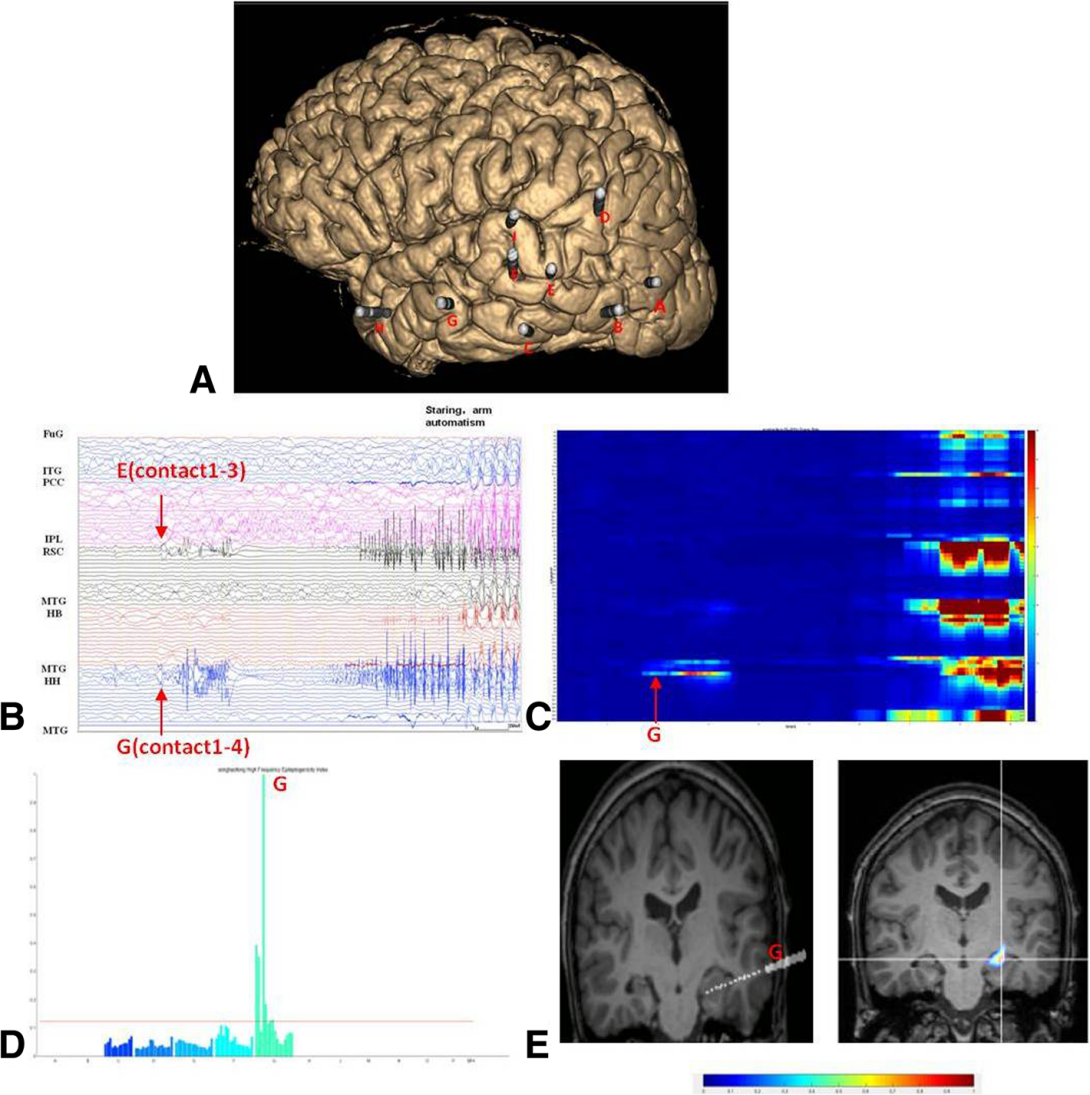
SEEG recordings of Patient 1. **a** Location of the SEEG electrodes shown on the 3D MRI image, The electrodes mainy explored the temporal occipital junction of left hemisphere. **b** From SEEG data, seizure started with a bursts of spikes in the head of hippocampus(internal leads of electrode G) and retrosplenial cortex(internal leads of electrode E);.**c** Quantification of the seizure onset confined to the electrode G. **d** The EI values quoted on the chart demonstrating maximal epileptogenicity in the electrode G. **e** EI analysis overlied MRI image, seizure onset was confined to the head of hippocampus. FUG:fusiform gyrus; PCC:posterior cingular cortex; IPL:inferior parietal lobule; RSC:retrosplenial cortex; MTG:middle temporal gyrus; HB:body of hippocampus; HH:head of hippocampus

**Fig. 3:**
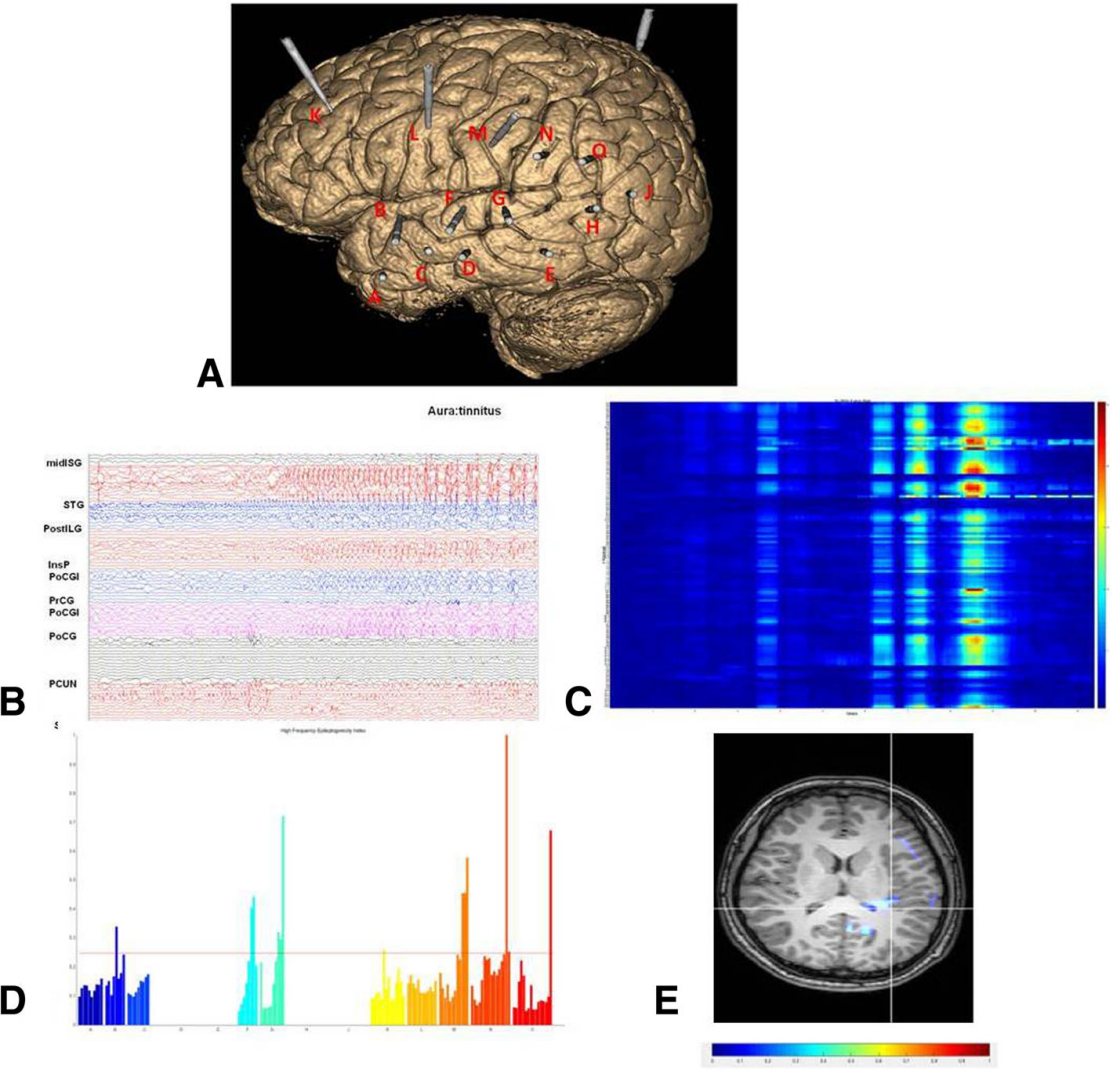
SEEG recordings of Patient 10.**a** Location of the SEEG electrodes shown on the 3D MRI image. **b** Seizure started with an onset of ictal discharge In either insular、temporal and frontal lobe within 2 s, not confined to a restricted area. **c** Quantification of the seizure onset were in Most electrodes simultaneously. **d** EI values quoted on the chart showing a wide range of epileptogenicity. **e** EI values overlied MRI image demonstrating several EZ zones. midISG:middle short gyrus;STG:superior temporal gyrus;postILG:posterior long gyrus;PoCGI:postcentral gyrus of insular; PrCG:precentral gyrus; PoCG:postcentral gyrus; PCUN:precuneus;SMG:supramarginal gyrus

For each seizure, the corresponding EI wascalculated. The objective was to Quantify and visualize seizure onset,that it can be shown prominantly. The EI is intended to highlight two features of SEEG signals recorded during the transition from preictal to ictal activity: (i)Energy ratio (ER), the redistribution of signal energy from lower frequency band (theta, alpha) toward higher frequency band (beta, gamma), ER = (Eβ + Eγ)/(Eα + Eθ), the behaviour of ER[n] on the SEEG signal during the transition from interictal to seizure activity was provided (Figs. 1c, 2c and 3c), (ii) the delay of appearance of these highfrequencycomponents in a given structure with respect to the first one [16]. Thus, after normalization, a numerical value between 0 and 1 is generated. An EI between 0 and 1 corresponds to secondary involvement of the considered brain structure.. In accordance with a previous study on epileptogenicity of developmental lesions, we set a cut-off EI value more than 0.4, which we considered a structure as highly epileptogenic [17]. we use a handy graphical user interface, the change points were detected automatically, EI was then computed in each visually defined seizure. Subsequently, we assigned the ictal onset zone defined by EI to an anatomical region, by superimposing it on MRI image, thus it allows a better presention of the anatomical location from which the seizure originates.

### Surgery and surgical outcome

All treatment decisions were made within a multi-disciplinary conference. SEEG exploration planning was performed based on the non-invasive data (including PET、MRI、scalp EEG etc), EI processing is based on SEEG signals,and epileptogenic zone for surgery should combine information including ictal onset (visually seen or EI), epileptogenic lesion, irritative zone, functional deficit zone all above: For Patient 1, information gained from EI and the MRI abnormality led to the definition of the epileptogenic zone, since one of the visually onset zone remote from the cortical lesion. The selective amygdalohippocampectomy was carried out on left side; For Three Patients (Pt no. 2,3,6), seizure onset defined by visual assessment was consistent with EI results, and a tailored resection was carried out; For Patient 4, although visual assessment were consistent with those of EI, SEEG RF-TC was chosen to protect occipital cortex function; For Patient 5, since EI suggested a larger ictal onset,we decieded to determine the extent of resection according to EI data; For four patients (Pst no. 7,8,9,10), the extent of epileptogenic zone defined by visually judgement exceeding the EI positive regions and MRI lesion, thus, a larger tailored resection was carried out.

Resective Surgery were performed in 9 patients. One patient (Pt4) underwent SEEG RF-TC at the end of the SEEG monitoring, without traditional resected surgery. There were no severe complications in our surgical series. Patients were followed up for at least 12 months. Engel scale was used for outcome classification. Class I: free of disabling seizures; class II: rare disabling seizures (“almost seizure-free”); class III: worthwhile improvement; and class IV: no worthwhile improvement [18]. Histopathologic examinations were performed according to the Consensus Classification proposed by International League Against Epilepsy (ILAE) [19].

## Results

### General features of patients

Demographic and clinical data of the patients were displayed in Table 1. The patients were listed according to the surgical outcomes (from Engel class I to Engel class IV) for better comparisons among groups. Ten patients (7 males and 3 females) underwent resective surgery or RF-TC after SEEG. Median age at viral infection was 11 years (6 months to 44 years), median age at first seizure was 11 years (20 months to 44 years), and median age at surgery was 19 years (10 years to 48 years).

**Table 1:** Main clinical characteristics of studied patients

Pt	Age at infection(y)	Age at first seizure(y)	Age at surgery(y)	Main semiology of seizure	Seizure frequency	MRI	PET-CT(hypometabolism)	Scalp VEEG	
								Interictal	Eegonset
1	≤5	≤5	6–10	1 loss of consciousness, eyes blinking, Bil arm automatism 2GTCS	1weekly 2 yearly	Bil- HS	L-T	L-T	L-Tδwave
2	11–15	11–15	21–25	staring, loss of consciousness,fumbling	weekly	normal	L-T	Bil-T (predominant left)	none
3	≤5	≤5	26–30	1eyes deviated to the R → GTCs 2 head burns, clenching(R), arm convulsion(R)	1 monthly 2 daily	abnormality in left center and parietal lobe	L-T、L-P	L-TP	none
4	≤5	≤5	11–15	1Aura:facial rubefaction→GTCS 2visual aura:colorful flashing,metamorphopsia	1 daily 2daily	Bil abnormal signal	Bil-P、 L-T	Bil-H	R-O
5	6–10	6–10	11–15	1 eyes、mouth deviated to the L 2staring, loss of consciousness	1 monthly 2 daily	normal	R-T	R-F, R-T	R-H
6	11–15	11–15	16–20	nausea, flustered	monthly	normal	L-T	Bil-T, Bil-F	R-F
7	11–15	11–15	11–15	1 mouth twitching, cyanotic lips 2 mouth twitching, L-arm tonic→GTCS	1 weekly 2weekly	normal	R-P、L-T	R-F(predominant left)	R-F
8	41–45	41–45	46–50	nausea, blinking,swallowing, R-arm dystonia →GTCS	weekly	normal	L-T	Bil-T, Bil-F	L-sphenoid
9	6–10	6–10	6–10	1 head deviated to the R → GTCS 2 staring	1 weekly 2daily	Bil-P abnormal signal	L-T、L-P	Bil-O (predominant left)	none
10	6–10	6–10	16–20	Aura:sound obscured, flustered→R arm numb、loss of consciousness→GTCS	monthly	normal	L-T	L-T, L-P, L-O	none

*Pt* patient, *y* year, *m* month, *L* Left, *R* right, *m* male, *f* female, *Bil* Bilateral, *GTCS* generalized tonic-clonic seizures, *HS* hippocampal scleros, *T* temporal, *P* Parietal, *F* Frontal, *O* occiutal, *H* hemisphere

Among 10 operated patients, 6 patients had normal MRI, 2 patients had bilateral abnormal signals (Pt no.4,9), 1 patient had bilateral hippocampal sclerosis (Pt no.1), and 1 patient had abnormality in left center and parietal lobe on MRI image (Pt no.3). hypometabolism of FDG-PET corresponded to a focal region in 6 patients (Pt no.1,2,5,6,8,10), and these hypometabolism were found in temporal lobe, while other patients had mild diffuse hypometabolism in multilobes.

All patients had a high seizure frequency, mainly manifested as nausea, facial rubefaction or cardiac frequency changes associated with partial tonic–clonic seizures, and other neurovegetative symptoms. Besides, 5 patients lost consciousness during the seizure or had symptoms of automatism, 4 patients had focal seizures, and 8 patients had secondarily generalized seizures. Neocortical temporal (auditory) and extratemporal (visual, somatosensory) auras were reported in 3 patients, respectively. The aura symptom had non-localizing or non-lateralizing values.

Scalp VEEG showed regional and generalized interictal epileptiform discharges, and the interictal discharges were focal in only 2 patients (Pt no.1,3), which were located in temporal lobe structure. The interictal abnormalities were bilateral in 5 patients (Pt no.2,4,6,8,9), multifocal in 1 patient (Pt no.10) and regional multifocal in 2 patients (Pt no.5,7). Seizure onset with voltage decrement was observed in 5 patients (Pt no.2,3,5,9,10), and the other patients had ictal onset with regional slow wave or discharges, not strictly confined to focal lobe.

### Surgical outcomes

All patients had follow-up at least 12 months. Among the 10 operated patients, 3 patients became seizure-free and reported a good persistent response (> 12 months) (Pt no.1,2,3), one of whom had selective amygdalohippocampectomy (SAH) and two of whom had surgery of lobectomy (Pt2, superior temporal gyrus including part of temporal pole; Pt3, postcentral gyrus). The efficacy of surgery achieved 30% in Engel class I (Pt no. 1,2,3); 5 patients achieved Engel outcome class II or III, and a worthwhile reduction of seizure or severity was achieved among these patients (Pt no. 4,5,6,7,8), and only 2 patients (Pt no. 9,10) did not get improvement after surgery (Engel class IV). Among the nonresponders to the surgery, 1 patient had subsequently been operated vagus nerve stimulation (VNS) and still got no improvement eventually.

### SEEG investigation

#### SEEG electroclinical findings

As shown in Table 2, all patients performed SEEG recordings, with a median number of 12 electrodes (8–15) and 148.8 contacts (86–180). The spontaneous seizures of a total of 99 patients (mean seizures for each patient: 9.9 ± 8.32; range: 4–32) were recorded and analyzed. For all the patients, 62.6% (*n* = 62) of seizures had a left onset, and 37.4% (*n* = 37) of seizures started from the right hemisphere.

**Table 2:** Results of SEEG recording

Pt	No.electrodes(contacts)	Side	Interictal spikes	Seeg onset zone(visually)	EI positive zone	Onset pattern (concomitant HFO)	Surgery (resected lobe)	Histopathology	Outcome(Engel) /FU(months)
1	9(126)	L	AM、Hi、RSC	AM、Hi、RSC	AM、Hi	bursts of spikes(HFO)	SAH	FCD Type III	I(28)
2	8(86)	L	AM、Hi、LAT-T	STS	STS	LVF(HFO)	STG、TP(L)	FCD Type IIIb	I(22)
3	15(172)	L	T、INS、P	POCG	POCG	LVF (HFO)	POCG(L)	FCD Type IIId	I(28)
4	18(232)	Bil	Bil-O	cuneus of O	cuneus of O	LVF(HFO)	Thermocoagulation of cuneus(R)	NA	II(23)
5	10(112)	R	STG、MTG、IFG、anterior INS	diffuse→MTG、HP	diffuse	voltage decrement	STG, MTG, anterior INS (R)	FCD Type Ia	II(43)
6	10(118)	R	MTG、HP、AM、IFG	Hi、AM	Hi、AM	slow wave(HFO)	Hi、AM, ATG (R)	FCD Type IIIa	II(41)
7	13(154)	R	T、INS、F、P	INS、OP of INS	INS	rhythmic discharges(HFO)	R-T、HP、AM、INS(R)	FCD Type Ia	III(24)
8	13(170)	L	TP、Hi、MTG、 anterior INS	AM、LAT-T、OP of INS	AM、LAT-T	rhythmic discharges	OP of INS、Hi、AM 、INS(L)	FCD Type III a	III (21)
9	12(138)	L	Hi、O、PCC、SPL	O、PCC、Hi	O、PCC、Hi	LVF (HFO)	HP、part of P、O(L)	FCD Type III d	IV(40)
10	12(180)	L	F、T、AG、INS	T、INS	T、INS	bursts of spikes(HFO)	T、Hi、AM、INS (L)	FCD Type III	IV(25)

*No* number, *AM* amygdala nuclei, *Hi* hippocampus, *RSC* retrosplenial cortex, *SAH* selective amygdalohippoeampectomy, *LAT-T* Lateral temporal, *STS* superior temporal sulcus, *TP* temporal pole, *T* temporal lobe, *INS* insula, *P* parietal lobe, *POCG* postcentral gyrus, *LVF* low voltage fast, *Bil* Bilateral, *O* occipital lobe, *STG* superior temporal gyrus, *MTG* middle temporal gyrus, *IFG* inferior frontal gyrus, *IFG* inferior frontal gyrus, *ATG* anterior temporal gyrus, *F* frontal lobe, *OP* operculum, *HFO* high freguency oscillation, *PCC* posterior cingular cortex, *SPL* superior parietal, *AG* angular gyrus, *FU* follow up, *NA* not applicable

The seizure-onset patterns showed no specificity and heterogeneity among patients, the distributed interictal recordings were not restricted to the focal region in all patients. As displayed in Table 2, the ictal onset zone (IOZ) originated from the restricted area in 5 patients, 2 of the patients from mesial temporal lobe (Pt no.1,6),and the remaining 3 were from superior temporal sulus (Pt no.2), postcentral gyrus (Pt no.3), and right lingual gyrus (Pt no.4). However, IOZ of other patients were started from the multiple brain areas. HFOs were detected and observed in eight patients, the other two had oscillations instead. We defined oscillations as segments of bursts with a frequency > 10 Hz, which is important not to confuse these oscillations with HFOs.

#### Other findings of SEEG

According to the correlation between the seizure-onset zone and efficacy of surgery, the patients were classified into two groups: Group 1 (with surgical outcome Engel class I and class II) includes 6 patients, they got totally seizure free (pt no.1,2,3; Engel scale, class I) or significant improvement (pt no.4,5,6; Engel scale, class II). The interictal discharges covered bilateral hemispheres from multiple lobes and the onset pattern of habitual seizure varied from HFO, LVF, poly spikes to slow waves. Meanwhile, we found that the seizures of most of the patients were started from the restricted area, especially in the seizure free group (pt no.1,2,3), and the onset of the discharges was located either in the mesial temporal structures (Pt1, hippocampus and amygdalar nucleus) or single gyrus (Pt2, superior temporal sulcus; Pt3, postcentral gyrus), then propagated to the ipsilateral or to contralateral structures. In the patients with palliative efficacy (pt no.4,5,6), two patients had confined onset (Pt4, right lingual gyrus; Pt6, hippocampus and amygdala nucleus), except that patient 5 whose SEEG onset originated with a voltage decrement first and then followed by discharges in focal area within seconds; Group 2 (with surgical outcome Engel class III and class IV) includes 4 patients (Pt no.7,8,9,10), they got worthwhile improvement or no improvement. Interictal discharges of SEEG involved different brain areas and seizure were started from multiple brain lobes, with a wide range of brain lesions.

### EI findings

**Fig. 4:**
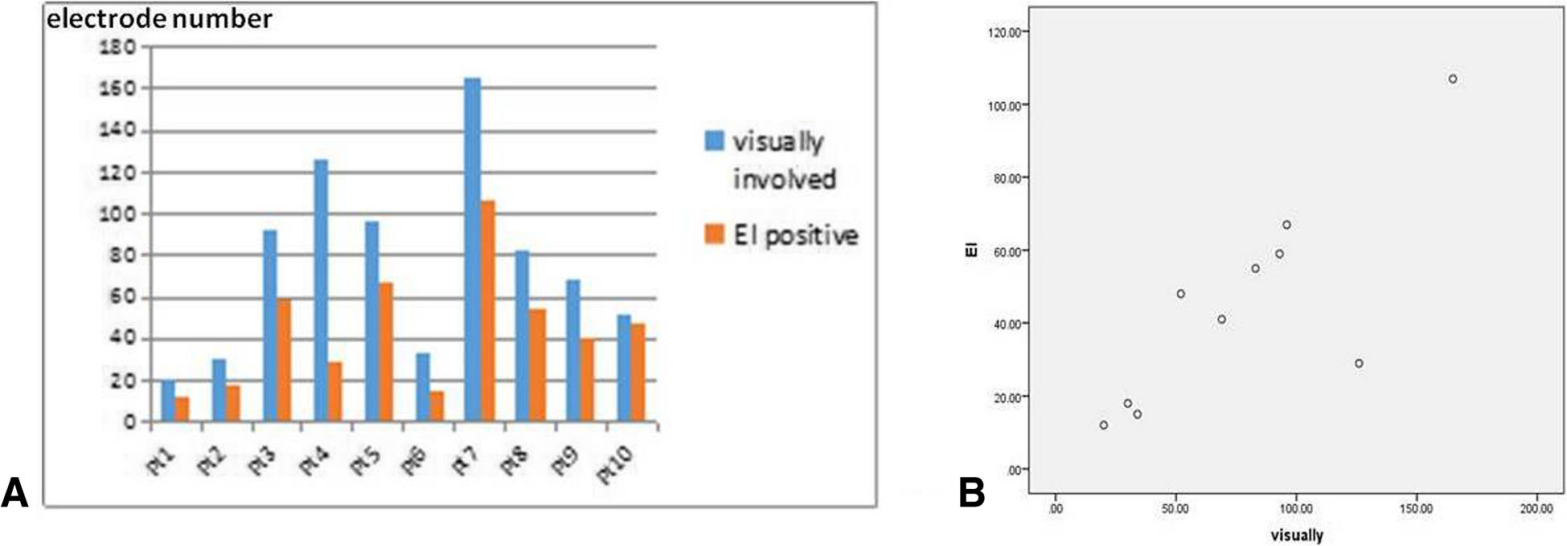
The number of electrodes visually involved and classified as epileptogenic by the EI in each patient (**a**) and they Were closely correlated (**b**)

The number of electrodes identified as epileptogenic visually were higher than EI (mean 77 visually involved electrodes vs mean 45 EI positive electrodes), but they correlated with each other closely (Spearman’s correlation: r = 0.794, *p* = 0.006) (Fig. 4). High values were restricted to the regional site in Group 1 and the highest EI values were consistent with their visual analysis (Figs. 1, 2 and 3). It was difficult to distinguish between the onset of seizure visually in some cases, and EI could help us to further clarify the starting range (Fig. 2). For example, three electrode contacts were identified as seizure onset by visual judgement, but not confirmed by EI for patient1.EI values were relatively diffuse in Group 2, which disclosed multifocal origin.

No completely contradictory results were found between EI results and visual analyses, which was shown on the MRI images (Figs. 1e, 2e and 3e). Based on this, we examined the outcome of the surgery determined by features of the EI value. Patients with good surgical outcome disclosed a high EI value restricted to the focal area. Changes in energy ratio of high frequencies (25–300 Hz; ER[n]) was observed in postcentral gyrus (Fig. 1e) and mesial temporal structures (Fig. 2e), which was depicted in a color scale. The patients with poor surgery outcome disclosed more complex patterns, with the most extensive range of ER increases, involving several electrodes (Fig. 3c-e).

## Discussion

The clinical presentation of VE is nonspecific, and patients with epilepsy after VE may usually develop intractable seizures [20–22]. In view of this, potentially epilepsy surgery should be considered as the treatment of epilepsy after VE [23–25]. Since VE has a great effect on the brain, the surgical outcome was usually unsatisfactory [26], and neuro surgeons were reluctant to touch this issue, this question was still underdiscussed. Patients undergoing surgery for drug-resistant epilepsy after VE constitute only a small minority in epilepsy surgery series. Nevertheless, some patients after VE do benefited from surgery. In a retrospective study [22], forty-two patients who suffered from epilepsy secondary to VE were included, and twenty-four patients were surgically treated. The postsurgical outcome was best in the unilateral temporal (UTLE) group and comparatively poor in the bilateral temporal (BTLE) and extratemporal and/or multifocal or generalized (ETMFE) group. In view of the above mentioned, we hoped to explore the feasibility of surgery in these patients, thus improving the quality of their lives.

Our study attached great importance on a highly selected population with intractable epilepsy following viral infections. Among the 10 operated patients, 3 patients got completely seizure-free and reported a good persistent response. The efficacy (Engel class I) of surgery achieved 30% in three of ten patients; 5 patients achieved Engel outcome class II or III, and a worthwhile reduction of seizure frequency was achieved among these patients, while only 2 patients did not get improvement after surgery, and we found a reduction in seizure frequency in two patients, which led to an increase in the quality of their lives. For these patients, surgical treatment will bring about palliation rather than treatment. From this point of view, a presurgical evaluation was necessary in patients with pharmacoresistant epilepsy after VE and they may get palliative or even curative surgical outcome by using SEEG, which makes the accurate localization possible.

It was widely accepted that younger age at surgery and a longer “silent period” between the acute infection and the onset was a good predictor for favorable surgical outcome [7, 26]. However, in our group, most of these patients had seizures in the occurrence of the first viral infection, there were seldom “silent period”. Whitley [27, 28] revealed that in the VE group, Focal seizures accounted for 65% and generalized seizures for 23%. In our group, 4 patients had focal seizures, and 8 patients had secondarily generalized seizures, thus we hypothesized that seizures appears to be more complicated and heterogeneous after encephalitis. According to interictal EEG data (scalp EEG), they had relatively restricted discharges especially in seizure free group. Scalp EEG onset is characterized by diffuse theta/delta activities and slow background, indicating the presence of a diffuse or multiregional damage of the brain. However, none of this can be a strong evidence of positioning, which explained the application of SEEG.

From MRI image, most of patients had normal image or non-specific abnormalities, two of them had bilateral abnormal signals, and only one had bilateral hippocampal sclerosis. Marks et al. reported that only 18% of patients with epilepsy after VE had imaging abnormalities [7], which was consistent with our conclusion. However, Trinke E’s research presented a different trend, the results of which were demonstrated that most of patients had regional and severe diffuse atrophy, with widespread T2 signal changes, more pronounced in the perisylvian and periinsular regions [22]. Some literature indicated that hippocampal atrophy in these patients had no responsibility for favorable postsurgical outcome, and more data are needed to further confirm it [3].

FDG-PET of 6 patients revealed areas of hypometabolism corresponding to focal regions, all of which were found in temporal lobe, which confirmed that encephalitis contributes to secondary damage to the temporal lobe. Interestingly, we found that those patients whose focal interictal discharges was consistent with FDG-PET (pt1,2,3), better surgical outcomes were achieved, which means that MRI negative and PET positive cases also confirmed the presence of the lesion.

The prognosis in our patients after surgical treatment is mainly depended on the seizure localization. Sellner and Trinka found that the vast majority of patients who had undergone temporal resections are characterized by post encephalitic epilepsy associated with mesial temporal sclerosis and are thus candidates for anterior temporal lobectomy with excellent results [25]. In these patients, great efforts should be made to figure out the clinical, EEG and imaging features, which may bring benefits to patients from epilepsy surgery. This result was consistent with previous clinical and experimental study, the application of HSV-1 to hippocampal cultures ex vivo could not only cause acute electrographic and clinical seizures, but also causes long-term excitability in hippocampal [29]. The virus especially HSV has the ability to replicate in neurons and glia cells. The inflammatory pathogenesis of VE is characterized by hemorrhagic-necrotizing features,and localized in the predilection sites within frontal and temporal lobe [30, 31]. All of these evidences provide an opportunity for surgery.

Since VE has a diffuse influence on the brain, performance of surgery becomes difficult, non-invasive are insufficient to lead straight to surgery. SEEG exploration is mandatory in the absence of a clear EZ or with diffuse lesion, especially for exploring deep seated structures.Even though we found that some patients had confined interictal discharges of scalp EEG, we still had to define the extension of EZ and its relationships with functional areas using SEEG. In a previous context, a sum of five patients underwent surgery following HSV-encephalitis, 2 of them became completely seizure-free and 3 had worthwhile improvement, implied that the resection of the leading focus or the focus generating the most disabling seizure type [32]. SEEG proved the theory in finding good surgical outcome in patients whose seizure onset started from mesial temporal lobe, which are in accord with the previous literature [32]. From our research, epileptogenicity in the patients with good outcome came not only from mesial temporal structure, but also from extratemporal area, especially from focal origin (Group1). Patients with seizure started from multiple brain lobes got worthwhile improvement or even no improvement at all (Group2). The correlation between the seizure-onset zone and efficacy mainly based upon SEEG can be a predictor for surgical outcome in the selection of the surgical indications for patients with VE.

EI estimation can be used to distinguish epileptogenic and non-epileptogenic structures, regardless of the underlying pathological substrate or the patient’s age [18, 33–35]. This method has been widely validated in the setting of epileptogenic neurodevelopmental disorders [36–38], but no relevant reports concentrated on EI in patients with epilepsy after VE. The objective of EI was to better characterize the EZ of each seizure. We found that there was excellent concordance between visual assessment and EI positive electrodes.This result suggests that epileptogenicity from SEEG signals can be characterized by a quantitative approach. Meanwhile, it could be an auxiliary tool to locate accurately in cases where visually seen onset is difficult to distinguish. We made the result more visible By assigning the EI-defined EZ to MRI image, which can further outline the dimension and distribution of the seizure onset. Except that, we also found a correlation between surgical outcome and EI values, that is, EI values were restricted to confined area in 5 patients who got curative or palliative outcome, demonstrating that despite the diffuse damage, the epileptic zone can be fairly concentrated in these patients. This also shows that if the ictal onset fulfills these criteria, it will achieve satisfactory surgical results accordingly.

## Conclusions

Successful tailored surgery may lead to a favorable outcome in patients with VE. In our study, we found that patients who got totally seizure free or had significant improvement had restricted seizure onset located either in the antero-mesial temporal structures or focal gyrus with the application of SEEG technology, which makes the accurate localization possible. EI is a quantitative measurement method, which can be used as a potential supplement to the visual analysis, and the validity of this technique is evaluated in the context of VE. EI values were high and restricted to confined area in those patients who got curative or palliative outcome. The limitation of the study is the relative small number of patient, making it impossible to prove the hypothesis statistically. Additionally, patients with epilepsy after VE are rarely recommended for surgical treatment due to the lesions are extensive and difficult to locate, not alone intracranial electrode implantation. Furthermore, to make a clear judgement, we need to set strict inclusion criteria, thus the included cases are fewer.

## Data Availability

Not Applicable due to patient confidentiality.

## Abbreviations

CT: Computed tomography
EEG: Electroencephalogram
EI: Epileptogenicity Index
EZ: Epileptogenic zone
FCD: Focal Cortical Dysplasia
SEEG RF-TC: SEEG-guided radiofrequency thermo-coagulation
SEEG: Stereoelectroencephalography
VEEG: Video-EEG
